# Association between proton pump inhibitor use and the risk of pancreatic cancer: A Korean nationwide cohort study

**DOI:** 10.1371/journal.pone.0203918

**Published:** 2018-09-12

**Authors:** In Cheol Hwang, Jooyoung Chang, Sang Min Park

**Affiliations:** 1 Department of Family Medicine, Gil Medical Center, Gachon University College of Medicine, Incheon, Republic of Korea; 2 Department of Biomedical Sciences, Seoul National University Graduate School, Seoul, Republic of Korea; 3 Department of Family Medicine, Seoul National University College of Medicine, Seoul, Republic of Korea; Catalan Institute of Oncology, SPAIN

## Abstract

**Purpose:**

Proton pump inhibitor (PPI) therapy causes hypergastrinemia, which could promote the development and progression of pancreatic cancer. Accordingly, this study aimed to investigate the association between PPI exposure and the risk of pancreatic cancer.

**Methods:**

We conducted a twelve-year longitudinal population-based study (2002–2013) using the Korean National Health Insurance Corporation claims database merged with national health examination data. The study cohort included 453,655 cancer-free individuals in January 2007 (index date). Incident pancreatic cancer was assessed throughout follow up until December 2013. The exposure to PPIs before the index date was assessed using a standardized Defined Daily Dose (DDD) system. We calculated the hazard ratios (HRs) and their 95% confidence intervals (CIs) for pancreatic cancer risk associated with cumulative PPI use using Cox proportional hazard regression models.

**Results:**

There were 3,086 cases of pancreatic cancer during the period of 2,920,000 person-years. PPI users exceeding 60 DDDs were at a higher risk of pancreatic cancer compared with non-users (HR, 1.34; 95% CI, 1.04–1.72). Subgroup analyses revealed that a significant association existed between PPI use and pancreatic cancer in low risk groups including individuals who were female, engaged in healthy lifestyle habits, and had no history of diabetes or chronic pancreatitis.

**Conclusion:**

Exposure to PPI appears to increase the risk of pancreatic cancer, independent of conventional risk factors.

## Introduction

Since their first introduction in the late 1980s, proton pump inhibitors (PPIs) have been widely used in clinical practice because they are generally well tolerated and highly effective [1]. The number of PPIs prescribed is rapidly increasing primarily due to their expanded applications including the treatment of gastroesophageal reflux disease, peptic ulcer disease, and functional dyspepsia, the eradication of *Helicobacter pylori* infection, and as a prophylaxis against the deleterious effects of non-steroidal anti-inflammatory drugs on the gastrointestinal tract. In addition, healthcare providers often prescribe PPIs for prolonged periods, sometimes lifetime of the patient, even in the absence of appropriate indications [2]. Thus, similar to other pharmacologic agents, there is a growing concern regarding the potential adverse effects of long-term PPI exposure [3].

Tumorigenesis is one of the major concerns among long-term PPI users. Gastric acid suppression creates a strong stimulus for gastrin production in G cells, which leads to increased plasma gastrin levels. Hypergastrinemia [4, 5] and hyperplasia of enterochromaffin-like cells [4, 6, 7] are commonly observed among long-term PPI users. *In vitro* and *in vivo* studies have shown that gastrin stimulates the growth of human pancreatic cancer cells through the gastrin receptor [8–10]. Notably, gastrin receptor antagonists prevent the growth of pancreatic cancer cells [8], and a gastrin inhibitor or antibody prolong survival in patients with pancreatic cancer [11, 12].

Although extensive basic research has focused on the carcinogenicity of PPIs in the pancreas, the relationship between PPIs and pancreatic cancer has not yet been established in humans. To the best of our knowledge, few epidemiologic studies [13–16], two of them utilizing the same databases just with different inclusion periods [13, 14], have been conducted to elucidate the associations between long-term PPI exposure and the risk of pancreatic cancer. A recent nested case-control study with an extended time period reported that long-term PPI use might increase the risk of pancreatic cancer in the UK population [13]. However, the study did not examine the dose-response relationship due to a lack of PPI dosing information; thus, reverse causation remained a possibility. Therefore, in this prospectively designed national cohort study involving a prescription database, we aimed to investigate the associations between PPI use and incidence of pancreatic cancer in the Korean population.

## Materials and methods

### Data source and study population

South Korea has a compulsory National Health Insurance system and the National Health Insurance Corporation (NHIC), as the single insurer, is responsible for managing this system, which offers universal coverage to nearly the entire population [17]. NHIC also provides biennial health examinations to all dependents over 40 years of age, which is used by 65.3% of the eligible subjects [18].

We used the data from a twelve-year standardized cohort (2002–2013), which were provided by the NHIC for research purposes under the stipulation that confidentiality be maintained. The NHIC claims database was merged with the national health examination database. We extracted the following information on individuals: age, sex, average insurance premium per month, comorbidities according to the *International Classification of Diseases code–10th Revision* (ICD-10) [19], and prescription data including drug name, dosage, and duration. For cancer diagnosis, we also used the Korean diagnosis-related group (DRG) claims for chemotherapy and surgery. Drug prescriptions were validated by cross checking pharmacy visits. We obtained height, weight, blood pressure, fasting glucose levels, and self-reported habits (tobacco use, alcohol consumption, and physical activity) from the health examination data nearest to the index date (January 1, 2007). Health-related habits did not contain the detailed information, such as amounts or forms of tobacco consumption, amounts or high frequency of alcohol consumption, and the types of physical activity. Prior high quality epidemiologic research has used the NHIC databases [20].

**Fig 1:**
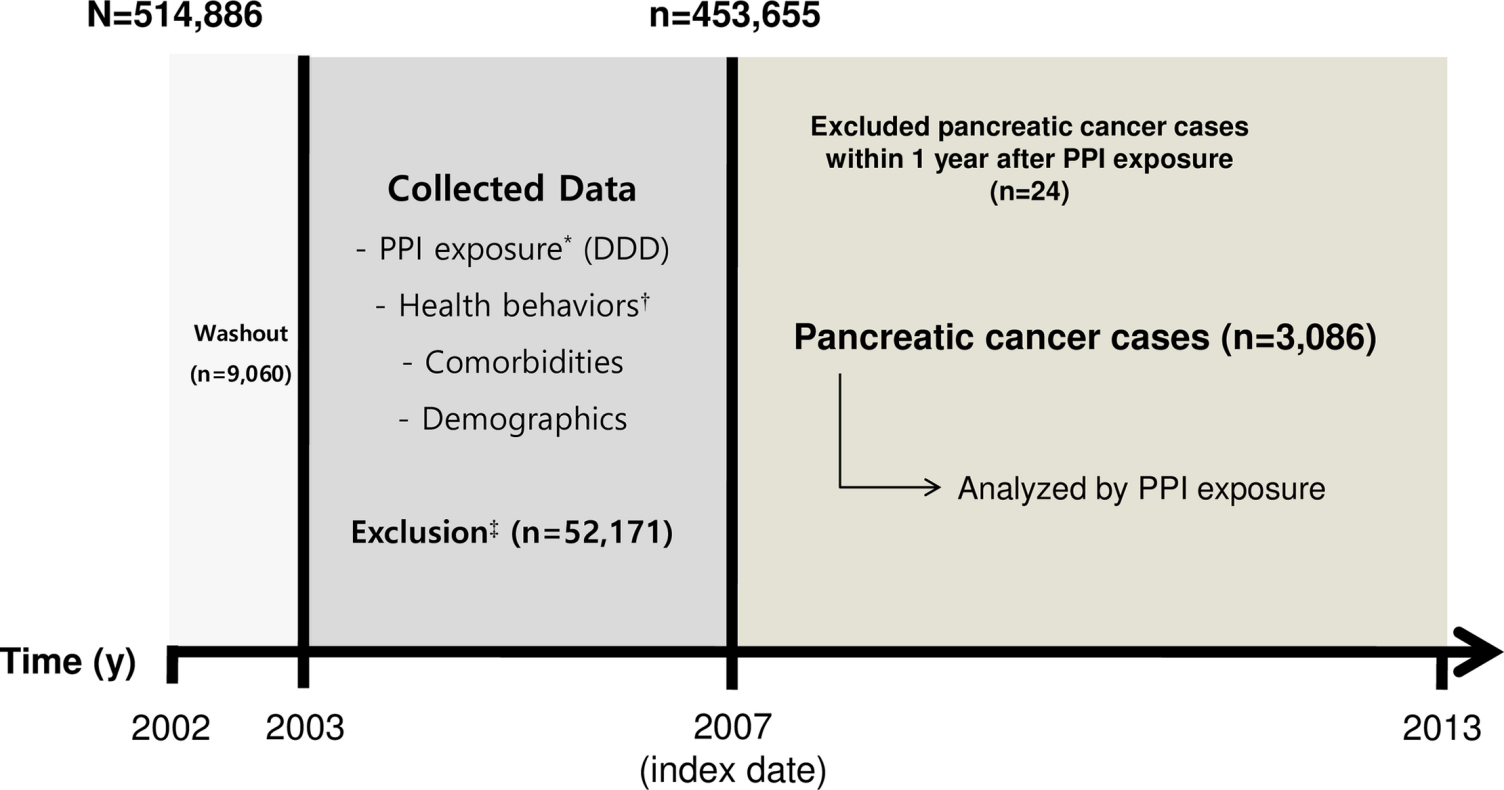
Study design and participant recruitment. DDD, Defined Daily Dose; PPI, proton pump inhibitor; NHIC, National Health Insurance Corporation. *Using the NHIC claims database. ^†^From national health examinations including body mass index, smoking status, drinking habits, and physical activity. ^‡^Patients with any cancer diagnosis by ICD-10 “C” code, with past medical history of cancer according to health check survey data, who died before the index date, or had missing non-survey health check-up variables were excluded from study. The exocrine type occupied the majority of pancreatic cancer cases (98.3%).

We identified individuals who were 40 years of age or older who received a health examination at least once between January 1, 2002 and December 31, 2006 (N = 514,886). To minimize the effect of PPI use prior to the study period, we excluded PPI users in 2002 (n = 9,060). We also excluded participants who had a history of cancer, as indicated by an ICD-10 “C” code or according to health examination survey data prior to the index date (January 1, 2007), and who had an any missing non-survey health check-up data (n = 52,171). To reduce protopathic bias resulting from cases of undetected pancreatic cancer that were prescribed PPIs based on symptoms, we excluded patients who were diagnosed with pancreatic cancer within 1 year after the index date (n = 24). We included a total of 453,631 participants in the analysis and observed the participants from the index date until the diagnosis of any cancer, death, or until December 31, 2013, whichever came first (Fig 1). The study protocol was approved by the Seoul National University Hospital Institutional Review Board (IRB number: E-1509-004-699), and the ethic committee waived the requirement for informed participant consent.

### Determination of incidence of pancreatic cancer

The primary outcome was a new diagnosis of pancreatic cancer, as indicated by an ICD-10 code in the nationwide claims database during the observation period. We defined pancreatic cancer as cases in which patients visited the hospital at least once with a C25 ICD-10 code and met any of the following criteria: (i) made at least three outpatient visits related to the C25 ICD-10 code, (ii) had three or more days of admission related to the code, (iii) received any curative cancer treatments claimed via the Korean DRG code “G60-Digestive Malignancy,” or (iv) died due to causes related to the code. The first date of diagnosis under the code was defined as the date of event for cases meeting those criteria. Patients that met the criteria but received a diagnosis other than cancer prior to the date of the event were not considered cases.

### Assessment of exposure and covariates

Information regarding all exposures and covariates during the period of four years prior to the index date was extracted. The primary exposure of interest was cumulative PPI use. We collected data on PPI prescriptions such as prescription dates, the daily dose, the number of days supplied, and number of pills per prescription. To indicate the PPI exposure, we used the Defined Daily Dose (DDD) system provided by the World Health Organization (WHO) Collaborating Centre for Drug Statistic Methodology [21]. The cumulative daily dose (in units of DDD) was computed, and subjects were categorized into three groups (no exposure, low exposure, and high exposure) based on a cut-off value of 60 DDDs.

We expressed comorbid conditions as a Charlson Comorbidity Index (CCI) score, which was calculated using the sum of the weighted scores of all comorbidities (e.g., cardiovascular, pulmonary, renal, and liver diseases) excluding diabetes and chronic pancreatitis [19]. Type 2 diabetes (T2D) was defined based on the ICD-codes or by a fasting blood glucose level of 126 mg/dL or higher in their health examinations. The body mass index (BMI) was calculated as the weight divided by the value of the height squared (kg/m^2^). For analysis, participants were classified into the following categories: BMI (<25.0, 25–29.9, or ≥30 kg/m^2^); frequency of physical activity (none, 1–2, or ≥3 times/week); smoking status (never, former, or current smoker); frequency of alcohol consumption (none, 1–2, or ≥3 times/week); CCI score (0, 1–2, or ≥3); and socioeconomic status (quartile 1–2 [low] or quartile 3–4 [high]).

### Statistical analysis

The primary analysis was a Cox proportional hazards analysis (Breslow method) to estimate hazards ratios (HRs) and 95% confidence intervals (CIs) for the association between PPI use and risk of pancreatic cancer.

We first identified the relevant factors associated with pancreatic cancer risk in our cohort and conducted sensitivity analyses (e.g., shifting the index date forward and backwards, extending the exclusion period up to 2 years, and confining to exocrine pancreatic cancer). We also looked at PPI use and pancreatic cancer risk according to subgroups of known risk factors for pancreatic cancer. In the stratified multivariable analyses, we reexamined the association between PPI use and the risk of pancreatic cancer among different subgroups. All analyses were performed using STATA Version 11.0 for Windows (STATA Corp., TX). We set the significance level at α = .05.

## Results

Table 1 shows the characteristics of the study population based on the PPI exposure levels. The results indicated that PPI exposure was associated with all variables (all *P*-values<0.001) such as age, gender, habits, comorbidities, and socioeconomic status.

**Table 1:** Characteristics of the study population by PPI exposure.

			Exposure level to PPI (%)		
		Total cohort	None	<60 DDDs	≥60 DDDs
%		(N = 453,611)	(n = 403,826)	(n = 44,075)	(n = 5,710)
Age, years					
	40–49	48.3	48.6	46.5	37.4
	50–59	28.1	27.9	30.3	32.1
	≥60	23.6	23.5	23.2	30.6
Gender, male		53.5	53.4	53.6	58.6
Body mass index, kg/m^2^					
	<25.0	65.1	65.1	65.3	62.3
	25.0–29.9	32.0	32.0	32.2	34.8
	≥30.0	2.9	2.9	2.5	2.9
Smoking status					
	Never	69.5	69.6	69.4	66.8
	Former	8.6	8.5	9.4	10.1
	Current	20.6	20.7	20.3	22.3
Alcohol consumption, drinks/week					
	None	72.7	72.7	73.1	73.0
	1–2	16.2	16.3	15.7	15.2
	≥3	10.5	10.5	10.7	11.1
Physical activity, times/week					
	None	52.5	52.5	52.0	53.0
	1–2	25.4	25.4	25.5	23.9
	≥3	21.3	21.2	21.9	22.2
Type 2 diabetes		10.7	10.7	10.8	13.6
Chronic pancreatitis		0.3	0.2	0.5	0.9
CCI* score					
	0	29.2	32.2	5.7	3.1
	1–2	54.5	53.1	67.1	61.1
	≥3	16.2	14.8	27.2	35.8
SES, low^†^		55.6	55.8	53.8	54.8

PPI, proton pump inhibitor; CCI, Charlson Comorbidity Index; SES, socioeconomic status.

*Including acute myocardial infarction, congestive heart failure, peripheral vascular disease, cerebral vascular accident, dementia, pulmonary disease, connective tissue disorder, peptic ulcer, liver disease, paraplegia, renal disease, severe liver disease, and HIV infection based on ICD-10 codes of hospital visits during years 2002 through 2006.

^†^By quartiles of insurance premium (Q1–2).

Table 2 lists the identified risk factors for pancreatic cancer in our cohort. A full adjusted Cox proportional model revealed that pancreatic cancer was more likely among individuals who were elderly (HR, 1.07 per 1 year; 95% CI, 1.06–1.07), male (HR, 1.41; 95% CI, 1.30–1.54), current smokers (HR, 1.25; 95% CI, 1.14–1.38), consumed alcohol more frequently (HR, 1.34; 95% CI, 1.20–1.49), or more experienced an increased number of comorbidities (*P*_trend_<0.001) including T2D (HR, 1.56; 95% CI, 1.38–1.77) and chronic pancreatitis (HR, 4.00; 95% CI, 2.96–5.42). Additionally, we found that individuals with increased exposure to PPIs experienced an increased risk of pancreatic cancer compared to individuals that were not exposed (HR, 1.32; 95% CI, 1.03–1.70).

**Table 2:** Adjusted HRs and 95% CIs for pancreatic cancer associated with PPI and covariates.

		Age-adjusted			Multivariate adjusted		
		HR	95% CI		HR*	95% CI	
Age (per 1 year)		—			**1.07**	**1.06**	**1.07**
Male		**1.54**	**1.44**	**1.66**	**1.41**	**1.30**	**1.54**
PPI exposure							
	None	1			1		
	<60DDDs	1.08	0.96	1.22	1.00	0.89	1.13
	≥60DDDs	**1.48**	**1.15**	**1.90**	**1.32**	**1.03**	**1.70**
Body mass index, kg/m^2^							
	<25.0	1			1		
	25.0–29.9	0.98	0.91	1.06	0.97	0.90	1.05
	≥30.0	1.09	0.88	1.34	1.04	0.84	1.28
Smoking status							
	Never	1			1		
	Former	1.00	0.87	1.15	0.98	0.85	1.12
	Current	**1.29**	**1.17**	**1.42**	**1.25**	**1.14**	**1.38**
Alcohol consumption, drinks/week							
	None	1			1		
	1–2	0.98	0.88	1.10	0.96	0.86	1.08
	≥3	**1.39**	**1.25**	**1.55**	**1.34**	**1.20**	**1.49**
Physical activity, times/week							
	None	0.99	0.90	1.08	0.97	0.89	1.06
	1–2	0.94	0.84	1.05	0.95	0.85	1.06
	≥3	1			1		
Type 2 diabetes^†^							
	No	1			1		
	Yes	**1.53**	**1.40**	**1.68**	**1.56**	**1.38**	**1.77**
Chronic pancreatitis^†^							
	No	1			1		
	Yes	**4.65**	**3.44**	**6.28**	**4.00**	**2.96**	**5.42**
CCI^‡^ score							
	0	1			1		
	1–2	**1.19**	**1.08**	**1.30**	**1.17**	**1.06**	**1.28**
	≥3	**1.55**	**1.39**	**1.72**	**1.46**	**1.30**	**1.63**
SES							
	Low^§^	1.05	0.98	1.13	1.03	0.96	1.11
	High	1			1		

HR, hazard ratio; CRC, colorectal cancer; PPI, proton pump inhibitor; CI, confidence interval; CCI, Charlson Comorbidity Index; DDD, Defined Daily Dose; SES, socioeconomic status.

*Using a Cox proportional hazards regression models with adjustment for all listed variables.

^†^Based on ICD-10 codes of hospital visits during years 2002 through 2006. Type 2 diabetes; two-time diagnosis or fasting blood glucose of 126 or higher.

^‡^Including acute myocardial infarction, congestive heart failure, peripheral vascular disease, cerebral vascultar accident, dementia, pulmonary disease, connective tissue disorder, peptic ulcer, liver disease, paraplegia, renal disease, severe liver disease, and HIV infection based on ICD-10 codes of hospital visits during years 2002 through 2006. Diabetes mellitus and chronic pancreatitis were not considered for CCI to prevent co-linearity.

^§^By quartiles of insurance premium (Q1–2).

There were 3,086 cases of pancreatic cancer in the entire cohort during the observation period of 2,920,000 person-years. In the sensitivity analysis, shifting the index date had an effect on the association between PPI exposure and the risk of pancreatic cancer. The statistical significance was dependent on the duration of follow-up, with a longer follow-up (or more cases) more likely to result in statistical significance (Table 3). Extension of the exclusion period or confining to exocrine pancreatic cancer did not affect the results. PPI exposure seemed to affect the risk of endocrine pancreatic cancer, but it did not reach the statistical significance. Table 3 presents the results of the subgroup analyses by the significant pancreatic cancer risk factors identified in Table 2. The significant effects of increased exposure to PPIs on the development of pancreatic cancer remained robust in low risk groups including females and individuals who practiced healthy lifestyle habits or had no history of T2D or chronic pancreatitis.

**Table 3:** Risk of PPI exposure for pancreatic cancer (case) development among various risk groups (reference: PPI non-user).

					<60 DDDs			≥60 DDDs			
			No. of cases	Person-years/10^5^	HR*	95% CI		HR*	95% CI		*P*_heterogeneity_^†^
*Shifting the index*, *year*											
	2006		3,537	33.7	1.10	0.97	1.25	**1.44**	**1.05**	**1.98**	0.122
	2007 (main)		3,086	29.2	1.00	0.89	1.13	**1.32**	**1.03**	**1.70**	**0.050**
	2008		2,625	24.7	1.02	0.91	1.14	1.01	0.90	1.13	0.904
*Extending the exclusion period*											
	1 year (main)		3,086	29.2	1.00	0.89	1.13	**1.32**	**1.03**	**1.70**	**0.050**
	2 years		2,155	29.2	1.01	0.87	1.16	**1.36**	**1.00**	**1.84**	0.084
*Types of pancreatic cancer*											
	All (main)		3,086	29.2	1.00	0.89	1.13	**1.32**	**1.03**	**1.70**	**0.050**
	Exocrine only		3,035	29.2	1.00	0.88	1.12	**1.30**	**1.00**	**1.68**	0.072
	Endocrine only		51	29.2	1.19	0.50	2.83	2.79	0.67	11.70	0.318
*Subgroup effect*											
	Age, years										
		40–49	761	14.6	0.94	0.74	1.21	1.55	0.89	2.69	0.105
		≥50	2,325	14.6	1.01	0.88	1.16	1.27	0.96	1.69	0.154
	Sex										
		Female	1,266	13.8	1.08	0.90	1.29	**1.68**	**1.16**	**2.44**	**0.036**
		Male	1,820	15.4	0.95	0.81	1.11	1.11	0.79	1.57	0.419
	Smoking status										
		Never	2,039	20.4	1.00	0.86	1.15	**1.47**	**1.09**	**1.98**	**0.023**
		Former or Current	1,000	8.5	1.01	0.82	1.24	1.05	0.65	1.70	0.884
	Alcohol, drinks/week										
		<1	2,167	21.2	0.99	0.86	1.14	**1.50**	**1.13**	**1.98**	**0.009**
		≥1	897	7.8	1.02	0.82	1.27	0.89	0.50	1.58	0.664
	Type 2 diabetes										
		No	2,520	26.2	0.97	0.86	1.11	**1.38**	**1.06**	**1.80**	**0.019**
		Yes	566	3.0	1.21	0.86	1.70	0.98	0.43	2.20	0.640
	Chronic pancreatitis										
		No	3,043	29.1	1.00	0.89	1.13	**1.30**	**1.00**	**1.69**	0.074
		Yes	43	0.07	—	—	—	—	—	—	—
	CCI score										
		0–1	1,551	18.1	0.97	0.78	1.20	1.23	0.71	2.13	0.430
		≥2	1,535	11.1	1.01	0.87	1.16	**1.35**	**1.01**	**1.79**	0.076

PPI, proton pump inhibitor; DDD, Defined Daily Dose; HR, hazard ratio; CI, confidence interval; BMI, body mass index; CCI, Charlson Comorbidity Index.

*Using Cox proportional hazards regression models with adjustment for all potential confounders listed in Table 2.

^†^Using a χ2 test for heterogeneity between log HR and CIs of <60 DDD and ≥60 DDD groups.

## Discussion

Pancreatic cancer is a highly lethal disease, mainly due to late diagnosis and/or early metastasis [22]. Because no standard program for screening high-risk patients currently exists, recognizing potential risk factors as well as conventional risks is important from an epidemiologic perspective. In this large population-based study, we found a positive association between PPI exposure and the risk of pancreatic cancer, which was robust in low risk subpopulations.

The mechanism by which PPI increases gastrointestinal malignancies is through the hormone gastrin, which is known to stimulate epithelial cell growth and to prevent apoptosis [23]. The normal feedback process that occurs between gastric acid and serum gastrin could lead to a chronic state of hypergastrinemia (i.e., PPI-induced hypergastrinemia). However, although animal studies have consistently demonstrated the carcinogenicity of PPIs [24], the association is less evident in humans [25, 26].

Another potential mechanism could be the alteration of the gut microbiome. Gastric acid is one of the barriers that prevent bacterial colonization in the upper gastrointestinal tract, which can influence the environment of the normal intestinal flora. PPI-induced hypochlorhydria could contribute to the proximal shifting of more distally colonized bacteria in the gut [27]. A prospective hospital-based study reported that the odds ratio of PPI users having a small intestine bacterial overgrowth (SIBO) compared with non-users was 2.46, independent of bowel motility [28]. To date, there is no direct evidence regarding the risk of pancreatic cancer in SIBO, but the fact that SIBO is common in patients with chronic pancreatitis suggests a potential biological link for some pancreatic cancers [29].

Despite theoretical mechanisms, very few epidemiologic studies have specifically examined the risk of pancreatic cancer among PPI users. Recently, large scale studies [13, 15, 16] demonstrated a positive association between long-term PPI use and the risk of pancreatic cancer, but the interpretation of their results was limited. Compared to those nested case-control studies, our cohort had a number of strengths. The DDD system from the verified prescription database could reliably assess PPI exposure, which enabled us to elucidate the dose-dependent response. Our sensitivity analysis, which expanded the follow-up duration, supported the strength of the association and indicated that further research involving a sufficient follow-up duration (or more cases) was warranted. In addition, to avoid an “immortal time bias”, we reconstructed a prospective cohort design, following up on the same calendar date for PPI users and non-users, and PPI use was treated as a time-dependent variable in Cox models. Likewise, the nested case-control design did not allow subgroup analyses by other covariates, because the follow-up time was truncated when the control reached the age of the case, and other exposure variables that were dependent on the follow-up time might be distorted [30].

Our subgroup analyses revealed that the significance of the association between PPI exposure and pancreatic cancer was confined to individuals with a low risk for pancreatic cancer, suggesting the importance of conventional risk factors. Conversely, the anticipated effects of well-known risk factors [31] on the development of pancreatic cancer supported the internal validity of our study. Our comprehensive data, which included national health examination results and the claims database, allowed us to assess confounders including chronic pancreatitis [32], T2D [33], history of smoking [34], and directly measured anthropometry [35] more extensively. Notably, no matter when these fixed risk factors were considered, higher exposure to PPI increased the risk of developing pancreatic cancer by 32% compared to individuals who were not exposed.

We acknowledge that our study had several limitations. First, our results cannot be generalized to other ethnicities due to the substantial geographical variation in known risk factors such as smoking and T2D [22]. Second, PPIs used during hospitalization were not considered. However, we believe that patients receiving PPIs by prescription represented most long-term users. Notably, our sensitivity analysis also highlighted the importance of the duration of follow-up over PPI exposure amount. Pancreatic cancer has a long latency period, which takes many years from the initiation to the progression to an advanced cancer stage [36]. Third, we could not capture detailed clinical data (e.g., smoking intensity or alcohol amount). In particular, the indications of PPI use, such as peptic ulcers and *Helicobacter pylori* infection, might be associated with an increased risk of pancreatic cancer [37, 38]. Adherence to prescribed PPIs that fell outside our analysis would also likely bias results. A large-scale hospital-based study or expansion of clinical trial cohort is needed to examine this association.

In conclusion, this study demonstrated an increased risk of pancreatic cancer among PPI users, and emphasized the conventional risk factors for pancreatic cancer. Our results might help physicians weigh the risks and benefits of PPI therapy: PPIs should be prescribed at the lowest effective dose over the shortest time period for patients with appropriate indications. Additional studies and collaborative basic research are necessary to confirm and further characterize our findings.
